# Examination of the breast asymmetry associated with adolescent idiopathic scoliosis using surface topography methods

**DOI:** 10.1186/1748-7161-8-S2-O39

**Published:** 2013-09-18

**Authors:** Alexandra Trovato, Amin Komeili, Lindsey Westover, Eric Parent, Marc Moreau, Samer Adeeb, Esteban Sepúlveda

**Affiliations:** 1 Department of Civil and Environmental Engineering, University of Alberta, Edmonton, Alberta, USA; 2 Department of Mechanical Engineering, University of Alberta, Edmonton, Alberta, USA; 3 Department of Physical Therapy, University of Alberta, Edmonton, Alberta, USA; 4 Department of Surgery, University of Alberta, Edmonton, Alberta, USA; 5 Facultad de Minas, Universidad Nacional de Colombia, Colombia

## Background

Breast asymmetry in females is significantly more common in adolescent idiopathic scoliosis (AIS) than in non-scoliotic females [1]. Researchers and clinicians currently use the “Cobb angle” from radiographs as the standard assessment method for scoliosis. Since exposure to radiation has been shown to increase the risk of cancer [2],radiography is not the safest method of assessment. Unlike surface topography (ST), radiographic assessments do not measure cosmetic deformities associated with AIS, which is very important to patients and has a psychological impact on the quality of life [3].

## Purpose

The objectives of this study were to observe the association between AIS and breast asymmetry using a 3-dimensional, markerless ST analysis technique, and to present a novel approach to analyze breast asymmetry that is associated with AIS. 

## Methods

Torso ST scans of 25 females with AIS (Cobb angle: 36.5°±14.0°, curve types: Lenke 1, 3, 5 and 6) were analyzed. The mean patient age was 15.4 ± 1.3 years (range: 13.5–17.5). At the time of the scan, two patients were pre-menarchal and the remaining had experienced menarche 1.9±1.1 years prior to the scan. The best plane of symmetry was found by mirroring the scan about the sagittal plane. The mirrored torso was fitted to the observed torso such that the average deviation between the torsos was minimized. The relative deviation between the mirrored and observed torso was measured and displayed as a deviation colour map (DCM). The DCMs were visually appraised, resulting in five types of breast asymmetry. 

## Results

Breast asymmetry was identified in all patients through a qualitative assessment. All had deviations exceeding 3mm between sides. The patients were classified into five distinct groups based on their pattern of breast asymmetry. 

## Conclusions and discussion

All of the patients presented breast asymmetry that could be categorized into five groups. Future work includes the correlation of breast asymmetry classification to the type and severity of the scoliosis curve. This ST analysis technique provides a non-invasive and objective method to assess breast asymmetry in patients with AIS. Further work is required to test the reliability of this classification. 
